# Assessing the utility of genomic selection to breed for durable Ascochyta blight resistance in chickpea

**DOI:** 10.1002/tpg2.70023

**Published:** 2025-03-31

**Authors:** Zibei Lin, Yongjun Li, Adnan Riaz, Shimna Sudheesh, Shahin Yazdifar, Judith Atieno, Sara Blake, Janine Croser, Joshua Fanning, Matthew J. Hayden, Sukhjiwan Kaur

**Affiliations:** ^1^ Agriculture Victoria Research, Department of Energy Environment and Climate Action Bundoora Victoria Australia; ^2^ South Australian Research and Development Institute Hartley Grove Urrbrae South Australia Australia; ^3^ The School of Agriculture, Food and Wine The University of Adelaide – Waite Campus Urrbrae South Australia Australia; ^4^ School of Applied Systems Biology La Trobe University Bundoora Victoria Australia; ^5^ Agriculture Victoria, Department of Energy Environment and Climate Action Horsham Victoria Australia

## Abstract

Ascochyta blight (AB) is one of the most devastating fungal diseases of chickpea (*Cicer arietinum* L.). Conventional breeding has focused on exploiting and introgressing major genes (qualitative effect) to improve AB resistance in released varieties. However, such approaches are time‐consuming and prone to the breakdown of disease resistance due to the fast evolution of AB pathogen. Genomic selection (GS) offers a promising alternative by predicting breeding values using genome‐wide single nucleotide polymorphisms (SNPs), regardless of major or minor effects. To our knowledge, this is the first study to develop and implement GS to improve AB resistance in chickpea. Over 4 years, 2790 chickpea lines, representing a broad range of germplasm collections primarily sourced from the Australian Grains Genebank, were evaluated for AB disease response in the field and in an outdoor pot‐based facility. Plants were genotyped with the Illumina multispecies pulse 30K SNP array, resulting in 23,239 high‐quality SNPs distributed across the genome. Intermediate‐to‐high genomic prediction accuracies (0.40–0.90) were achieved across validation scenarios. Bayesian modeling identified six major QTL explaining 33% of the genetic variance for AB resistance, with the remaining variance explained by minor effect genes. Using genomic estimated breeding values (GEBVs), 462 lines of the 2790 lines were predicted to have higher resistance compared to the released check varieties, revealing the potential of further improvement of AB resistance for the industry. The desirable genomic prediction accuracy obtained in the study supports the applicability of GS to breed for AB resistance in chickpea.

## Core Ideas

- This is the first study that developed and implemented genomic selection (GS) to breed for Ascochyta blight (AB) resistance in chickpea.
- Desirable genomic prediction accuracy was achieved, supporting the applicability of GS to breed for AB resistance.
- GS is being implemented to breed for AB resistance materials for chickpea breeders.

**Abbreviations**
AB: Ascochyta blight
AGG: Australian Grains Genebank
CCDM: the Centre for Crop and Disease Management, Australia
FLIP: the Food Legume Improvement Program
*Fst*
: fixation indices
GBLUP: genomic best linear unbiased prediction
GEBV: genomic estimated breeding value
GRM: genomic relationship matrix
GS: genomic selection
gVar: genetic variance
HT: herbicide tolerance
LD: linkage disequilibrium
MAF: minor allele frequency
PCA: principal component analysis
QTL: quantitative trait loci
SNP: single nucleotide polymorphism
UWA: The University of Western Australia

## 1 INTRODUCTION

Chickpea (*Cicer arietinum* L.) is one of the most important cool‐season legume globally in terms of planting area and production, serving both human and animal consumption needs. The global chickpea market was valued at AUD 21 billion in 2022 and is projected to increase to AUD 31 billion by 2032 (Global Market Insight, 2023). In addition to being a valuable protein source, chickpea fixes atmospheric nitrogen through rhizobial association and supplies organic nitrogen to the successive crop, provides monocot weed management options, and can be used to break cereal disease cycles in broadacre systems. However, the productivity of chickpea can be significantly impacted by various biotic and abiotic stresses. Among the biotic stresses, Ascochyta blight (AB) is the most devastating disease, caused by the fungus *Ascochyta rabiei* (syn. *Phoma rabiei*). The fungus can infect all aboveground parts of the plant and is most severe when cool, cloudy, and humid weather occurs during the crop growing season (Moore et al., 2015). AB infection leads to a range of symptoms including lesions on leaves, stem, and pods, resulting in a significant reduction in vigor, seed quality, and yield. The grain yield losses due to AB in chickpea have been reported to be up to 96% in susceptible varieties and 64% in moderately susceptible varieties, highlighting the importance of breeding for AB resistance (Fanning et al., 2022; Markell et al., 2008).

Developing resistant varieties remains the most cost‐effective and practical approach for disease management in chickpea. Extensive research has identified multiple qualitative trait loci (QTL) contributing to AB resistance in various experimental settings including glasshouse and field trials using cultivated germplasm (Deokar Sagi, & Tar'an, 2019; Stephens et al., 2013; Sudheesh et al., 2021). However, narrow genetic diversity within cultivated germplasm has limited the identification of useful sources of resistance (Ghaffari et al., 2014; Jamalabadi et al., 2013). To address this, novel resistance could be sourced from the broader chickpea gene pool, including wild relatives and landraces (Jha et al., 2022; Newman et al., 2021, etc.). Though conventional breeding has delivered some success in enhancing the level of AB resistance in cultivated germplasm through the deployment of major resistance genes (Stephens et al., 2013), the rapid evolution of the AB pathogens (Bar et al., 2021; Deokar, Sagi, Daba, et al., 2019) often results in the fast breakdown of resistance in released varieties. This highlights the need for the continuous monitoring of pathogen populations and the deployment of new breeding strategies that can better withstand pathogen evolution. Such strategies could include exploiting both minor (quantitative) and major (qualitative) effect genes to achieve improved and durable resistance.

Genomic selection (GS) has gained popularity in plant breeding over the past decade (Crossa et al., 2017; Jighly et al., 2021; Lin et al., 2021; Windhausen et al., 2012). GS is a statistical method that uses DNA markers with genome‐wide distribution to simultaneously account for qualitative (major genes) and quantitative (minor genes) marker effects to estimate the genetic potential of selection candidates. From a model trained by the reference population with both phenotypes and genotypes, genomic estimated breeding values (GEBVs) of selection candidates can be obtained without directly measuring the candidates’ phenotypes. GS has been shown to be especially beneficial in breeding for complex traits that have low heritability and/or are difficult to measure. GS can improve the rate of genetic gain per unit time by improving selection accuracy and intensity, while reducing generation intervals (e.g., Gebremedhin et al., 2024; Lin et al., 2016, etc.).

AB resistance in chickpea has previously been reported as a moderately complex trait influenced by both major effect genes and numerous minor effect genes (Daba et al., 2016; Sudheesh et al., 2021). Hence, it is anticipated GS could be an efficient approach to breed for improved AB resistance in chickpea through the combining of both major and minor effect resistance genes. To test the efficacy of GS, we assessed a large and diverse set of *Cicer* germplasm including landraces and wild *Cicer* introgression lines for AB disease response under field and pot‐based conditions. We then investigated the genetic architecture for the observed AB resistance and examined the prediction accuracy of GEBVs for the trait to determine the applicability of GS for developing chickpea varieties with more durable resistance to AB disease.

## 2 MATERIAL AND METHODS

### 2.1 Genotypic data

A total of 2790 chickpea lines were obtained from multiple sources (Table 1). These comprised Australian released cultivars; breeding lines from Chickpea Breeding Australia (CBA), the Food Legume Improvement Program (FLIP), and Vavilov collections sourced from the Australian Grains Genebank (AGG, Horsham, Victoria); F4 and F5 families derived from *Cicer arietinum* x *C. arietinum* subsp. *reticulatum* (L.) Ladiz. crosses developed by University of California, Davis (UC Davis), and the Centre for Crop and Disease Management (CCDM) Australia, as well as herbicide‐tolerant mutant lines (HT) and cultivars and historical lines derived from The University of Western Australia (UWA)‐led Council of Grain Growers Organisation Pty Ltd (COGGO) funded chickpea AB breeding project. The wild introgression lines used in this study were developed in von Wettberg et al. (2018) by crossing elite cultivars to several wild *Cicer* species from Turkey.

**TABLE 1:** Sources and description of the 2790 lines.

Collection	Type	No. of lines
CBA	Breeding lines	396
CCDM	Wild introgression lines	443
FLIP	Pre‐breeding materials from the ICARDA	1212
HT	Herbicide‐tolerant mutants	7
UC Davis	Wild introgression lines	193
UWA	Cultivars/historical germplasm	15
Vavilov	Cultivars/landrace	524
	Total	2790

Abbreviations: CBA, Chickpea Breeding Australia; CCDM, Centre for Crop and Disease Management; FLIP, Food Legume Improvement Program; HT, herbicide tolerance; ICARDA, International Center for Agricultural Research in the Dry Areas; UC Davis, University of California, Davis; UWA, University of Western Australia.

All lines were genotyped using the Illumina multispecies pulse single nucleotide polymorphism (SNP) assay described in Gebremedhin et al. (2024). A total of 4449 SNPs were used to impute 765,803 whole‐genome sequence SNP using Beagle v4.1 (Browning & Browning, 2013) and aligned by CDC Frontier version 10 (Varshney et al., 2013). Following stringent filtering based on a linkage disequilibrium (LD) sliding window size of 200, R^2^ of pairwise LD < 0.99, R^2^ of imputation > 0.8, and MAF > 0.05, a total of 23,239 high‐quality genome‐wide SNP remained that were used for further analysis.

**Core Ideas**

- This is the first study that developed and implemented genomic selection (GS) to breed for Ascochyta blight (AB) resistance in chickpea.
- Desirable genomic prediction accuracy was achieved, supporting the applicability of GS to breed for AB resistance.
- GS is being implemented to breed for AB resistance materials for chickpea breeders.

### 2.2 Phenotypic data

Phenotyping of the 2790 chickpea lines was conducted both in field trials and in a pot‐based assay (Table S1) over 3 years (2020–2022). For details of the phenotyping methods, see Atieno et al. (2024). In brief, field experiments were conducted by Agriculture Victoria Research at Horsham, Victoria, Australia (−36.74°S, 142.11°E) from 2020 to 2022. A total of 10 seeds were sown in a 1‐m single row in a partial replicate design, from late June to early July each year. To facilitate nitrogen fixation, each field plot was treated with 5 kg per hectare of Rhizobium inoculant (Nodulator Chickpea granular inoculant, BASF) along with the seed. To maintain weed and insect pest‐free conditions, appropriate herbicides and insecticides were applied. Field plots were inoculated at the four‐node stage (late August to early September) with infected stubble from naturally infected field plants. The pot‐based screenings were conducted outdoor within an irrigated shade house at the South Australian Research and Development Institute, Adelaide, Australia (34.9670°S, 138.6360°E). Plants grown in pots were inoculated with single isolate of the target pathogen (isolates collected from southern and northern regions). In addition, phenotypes collected from the previous projects (2016–2019) were also included to expand the training population for GS. Disease assessment was performed by evaluating the percentage of stem breakage (1%–100%) between October and November (flowering stage) before disease progression was halted by warm temperatures.

### 2.3 Data analysis

#### 2.3.1 Genetic diversity between collections

To assess the genetic diversity of the germplasm, principal component analysis (PCA) was conducted. Principal components (PC) were estimated by fitting the genotypes of all lines to “prcomp” (a default function of R [R‐Core‐Team, 2022]), of which PC1 and PC2 were plotted using the “ggplot2” R‐package (Wickham, 2016). Population differentiations were estimated by calculating fixation indices (*Fst*) between pairs of collections (Table 1) using the “snpReady” R package (Granato & Fritsche‐Neto, 2018).

#### 2.3.2 Pre‐processing of the raw phenotypes

Using the data collected from pot‐based assay, genetic correlations were calculated to assess the relationship between genotypic responses to different isolates used (Table S1). Due to the high correlation observed (see Section 3 for detail), stem breakage percentage measures from different isolates were treated as a single trait, with data from the pot‐based screens averaged across isolates per genotype. For the field data, a linear mixed model was fitted within each year to account for spatial heterogeneity (i.e., row and column effects). Variance components were estimated using REML, and BLUEs were calculated for the fixed genotype effects using ASReml‐R 4 (Butler et al., 2018) with the following model:

(1) $$y=\mathrm{Xb}+Z_{c}c+Z_{r}r+e,$$

where y is the vector of the response variable (stem breakage in percentage per year); X is the incidence matrix of fixed effects, and b is a vector of fixed effects (in this case, the population mean, replicates and the individual IDs); $Z_{c}$ and $Z_{r}$ are incidence matrices of random factors, and c and r are vectors of the column and row effects; e is the error term distributed as [0, $\sigma_{e}^{2}$ **Σ_c_** (ρ**_c_**)⊗**Σ_r_** (ρ**_r_**)], where ⊗ is the Kronecker product, $\sigma_{e}^{2}$ is the error variance, and **Σ_c_** (ρ**_c_**) and **Σ_r_** (ρ**_r_**) are the matrices of correlations within columns and within rows, respectively, both modeled by using auto‐regression structure (AR1).

#### 2.3.3 Heritability

Heritability of the percentage of stem breakage from the field and the pot‐based screening was estimated using the following genomic best linear unbiased prediction (GBLUP) model:

(2) y=Xb+Zg+e,

where y is the vector of the response variable (field or pot‐based); b is a vector of fixed effects (the population mean), with its designed matrix X; g is a vector of the random additive genetic effects distributed as $N(0,\sigma_{g}^{2}G)$, where $\sigma_{g}^{2}$ is the additive genetic variance and G is the genomic relationship matrix (GRM) developed using SNP genomic data (VanRaden, 2008) with its designed matrix Z; and e is the error term distributed as $N(0,\sigma_{e}^{2}I)$, where $\sigma_{e}^{2}$ is residual variance and I is the identity matrix. The narrow sense heritability was calculated as: $h^{2}=\frac{\sigma_{g}^{2}}{\sigma_{g}^{2}+\sigma_{e}^{2}}$.

#### 2.3.4 Bivariate analysis with GBLUP

Two bivariate analyses were used to investigate correlations in the datasets. The first analysis estimated the genetic correlations between genotypic responses to different isolates. In this analysis, trait 1 represented the stem breakage caused by one isolate, while trait 2 represented the stem breakage caused by another isolate. The purpose was to assess the degree of genetic similarity or dissimilarity between genotype responses to different isolates. The second bivariate analysis focused on estimating the genetic correlation between the stem breakage data measured in the field and in the pot‐based screen. Trait 1 in this analysis represented the field phenotypes, and trait 2 as the disease scores obtained from the pot‐based screens. The bi‐variate model used was:

(3) y=Xb+Zg+e,

where y is the vector of phenotypes as described above; b is a vector of fixed effects (e.g., the population mean, fitting trials of isolates when estimating between isolates, or data sourced when estimating between field and pot‐based), with its designed matrix X; g is a vector of the random additive genetic effects, distributed as $N(0,G_{0}⊗G)$ with $G_{0}=\left[\begin{array}{cc} \sigma_{g_{1}}^{2} & \sigma_{g_{12}}\\ \sigma_{g_{12}} & \sigma_{g_{2}}^{2} \end{array}\right]$, $\sigma_{g_{1}}^{2}$ and $\sigma_{g_{2}}^{2}$ are the additive genetic variances for traits 1 and 2, and $\sigma_{g_{12}}$ is the additive genetic co‐variance between traits 1 and 2, and G is the GRM as described in the section above; and e is the error term distributed as $N(0,R_{0}⊗I)$ with $R_{0}=\left[\begin{array}{cc} \sigma_{e_{1}}^{2} & \sigma_{e_{12}}\\ \sigma_{e_{12}} & \sigma_{e_{2}}^{2} \end{array}\right]$ where $\sigma_{e_{1}}^{2}$ and $\sigma_{e_{2}}^{2}$ are the residual variances for traits 1 and 2, respectively, $\sigma_{e_{12}}$ is the residual co‐variance between traits 1 and 2, and I is the identity matrix as described in the section above. The genetic correlation between traits was calculated as $r_{g}=\frac{\sigma_{g_{12}}}{\sqrt{\sigma_{g_{1}}^{2}*\;\sigma_{g_{2}}^{2}}}$.

### 2.4 Cross‐validation

Multiple validation scenarios were implemented to evaluate genomic prediction accuracy: (1) cross‐validation between isolates within the pot‐based data, where GS models were trained using the response of one isolate to predict the response of another isolate; (2) fivefold cross‐validation using unique genotypes without any overlap between the training and the validation population; (3) leave‐one‐year‐out validation on the field data, with “year” fitted as fixed effect to account for environmental variability; and (4) cross‐validation using the field data for model training to predict the pot‐based data and vice versa. Overlapping lines between the training and test populations were removed from the training, except when predicting field from pot‐based and vice versa as that would have removed all lines. GEBVs were calculated for all genotypes evaluated in the study. The prediction accuracies were assessed using the Pearson correlation between GEBVs and phenotypes.

### 2.5 Identification of major QTL for AB resistance

Bayesian modelling was undertaken to calculate marker effects for the identification of major QTL associated with AB resistance. In brief, phenotypic scores obtained from field trials conducted over 3 years (2020–2022) were fitted to the same model as described in Section 2.3.3, where “year” was fitted as fixed effect to account for environmental variability. The adjusted phenotypes were then fitted to BayesR (Breen et al., 2022), assuming 94%, 4.9%, 1%, and 0.1% of SNP explained 0%, 0.0001%, 0.001%, and 0.01% of genetic variances, respectively. The actual genetic variance (gVar) explained by each marker was estimated using the equation:

(4) $$\mathrm{gVar}=2\times p\times q\times \sigma^{2},$$

where *p* and *q* are the frequencies of the reference and alternative alleles per marker and *σ* is the marker effects estimated by BayesR. The percentage of genetic variance (%gVar) explained per marker was calculated as gVar / Σ(gVar). The estimated marker effects were plotted using “*ggplot2*” in R (Wickham, 2016). QTL were defined as the markers that explained >1% gVar. Due to the high similarity between the two systems in terms of revealing genotypic responses to AB, only the outcomes from field data were shown for QTL identification (for more details, see Sections 3 and 4).

### 2.6 Estimation of GEBVs from diverse collections and comparison to the released check varieties

Bivariate GBLUP model (the same as described in Section 2.3.4) was trained using all field and pot‐based data to predict GEBVs for the 2790 lines. The performance of lines from the diverse collections was measured and compared to the released check varieties in this project based on the GEBVs.

## 3 RESULTS

### 3.1 Genetic diversity, heritabilities, and genetic correlations

**FIGURE 1:**
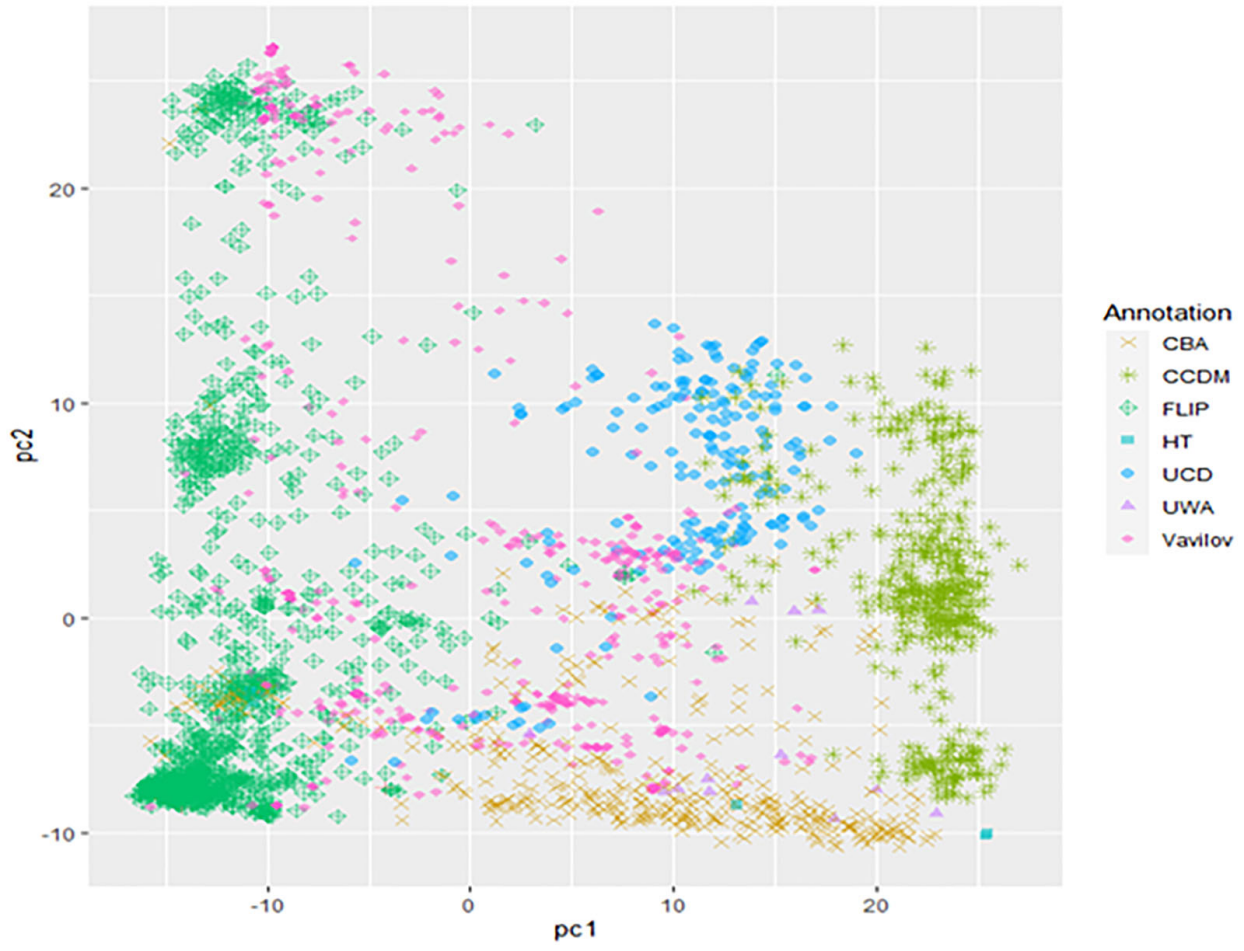
Genetic diversity of the 2790 lines from the seven collections, with 54% variance explained by PC1 and 26% by PC2. CBA, Chickpea Breeding Australia; CCDM, Centre for Crop and Disease Management; FLIP, Food Legume Improvement Program; HT, herbicide tolerance; UCD, University of California, Davis; UWA, University of Western Australia.

PCA revealed distinct separations among the different germplasm collections (Figure 1). Wild *Cicer* introgression lines developed by CCDM and UC Davis showed a degree of overlap, while the FLIP lines were clearly distinguished from other collections, except for some overlap with the Vavilov collection. The CBA breeding lines overlapped with the FLIP, Vavilov, CCDM and UC Davis collections. The analysis showed a small‐to‐moderate level of population differentiation across the collections as evidenced by an *Fst* value of 0.197 for the overall collection. The *Fst* values for among collections are shown in Table S2.

The numbers of lines overlapping between trials ranged from 1 to 531 (Table S3). Data analysis revealed high narrow sense heritability for both the field data (0.82) and the pot‐based data (0.69). There was also a strong genetic correlation (0.91) between field trials and pot‐based assay. With the exception of isolate TR8102, the majority of the isolates showed strong genetic correlations with other isolates (Table 2).

**TABLE 2:** Genetic correlations between responses to isolates estimated from the pot‐based data.

	16CUR018	TR8102	TR9571	AR0128	AR0226
16CUR018	–				
TR8102	0.84 ± 0.04	–			
TR9571	0.89 ± 0.03	0.27 ± 0.19	–		
AR0128	0.81 ± 0.05	0.04 ± 0.14	0.84 ± 0.08	–	
AR0226	0.90 ± 0.03	0.13 ± 0.16	0.27 ± 0.19	0.99 ± 0.01	–

### 3.2 Cross‐validations

Moderate‐to‐high genomic prediction accuracy was achieved across all validation scenarios. The accuracy of predicting one isolate using the other isolate as training set was found to be moderate to high (0.40–0.90; Table 3). High accuracy was also achieved for the field data using fivefold random cross‐validation (0.87 ± 0.01) and the leave‐one‐year‐out method (0.73–0.84). Similarly high accuracy was observed when the model was trained using field data to predict the pot‐based data (0.63) and vice versa (0.81).

**TABLE 3:** Genomic prediction accuracy between isolates from the pot‐based data, pooled across years which data available.

Training population	Validation				
	16CURY018	TR8102	TR9571	AR0128	AR0226
16CURY018	–	0.90	0.67	0.69	0.78
TR8102	0.56	–	0.40	0.52	0.52
TR9571	0.51	0.51	–	0.54	0.54
AR0128	0.55	0.76	0.67	–	0.66
AR0226	0.67	0.83	0.60	0.76	–

### 3.3 Identification of major QTL for AB resistance

**FIGURE 2:**
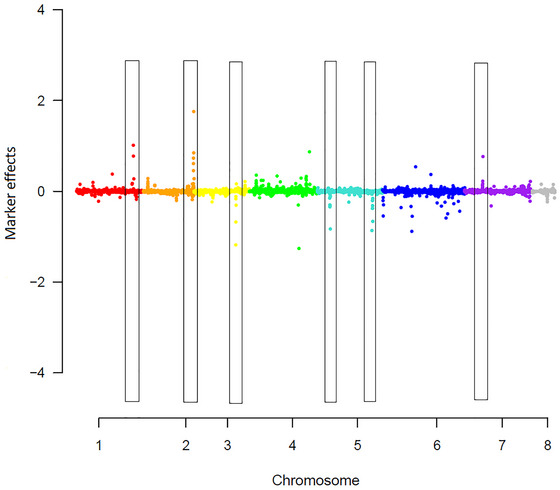
Manhattan plot of marker effects across the eight chickpea chromosomes, with identified qualitative trait loci (QTL) highlighted in rectangles, estimated using pulled data from the field trials across 2020–2022.

Marker effects across the genome were estimated by BayesR (Figure 2). Six QTL explaining a total of 33% of the genetic variance were identified using the field data (Table 4). QTL analysis was conducted using both field and pot‐based assays, with most QTL overlapping. Given the higher relevance of field results for breeding practice, the study focused on discussing the field data. Among these, the QTL on chromosome 2 (chr2_36033767) explained the largest proportion of the genetic variance (17.7%), followed by two QTL on chromosomes 1 and 5 (chr1_40754110 and chr5_39193379) with each explaining 3.8% of the genetic variance. The remaining three QTL (chr3_30047057, chr5_9238098 and chr7_13041287) contributed 7.7% of the genetic variance.

**TABLE 4:** Six qualitative trait loci (QTL) regions identified through BayesR analysis.

Chr	Start	End	Peak position	Percentage of genetic variance (%gVar)
1	40,696,866	41,136,378	40,754,110	3.8
2	35,932,089	36,069,106	36,033,767	17.7
3	29,694,732	31,498,390	30,047,057	1.9
5	8,826,941	9,408,172	9,238,098	2.6
5	39,193,379	39,817,464	39,193,379	3.8
7	12,853,075	13,041,287	13,041,287	3.2

### 3.4 Estimation of GEBVs from diverse collections and comparison to the released check varieties

**FIGURE 3:**
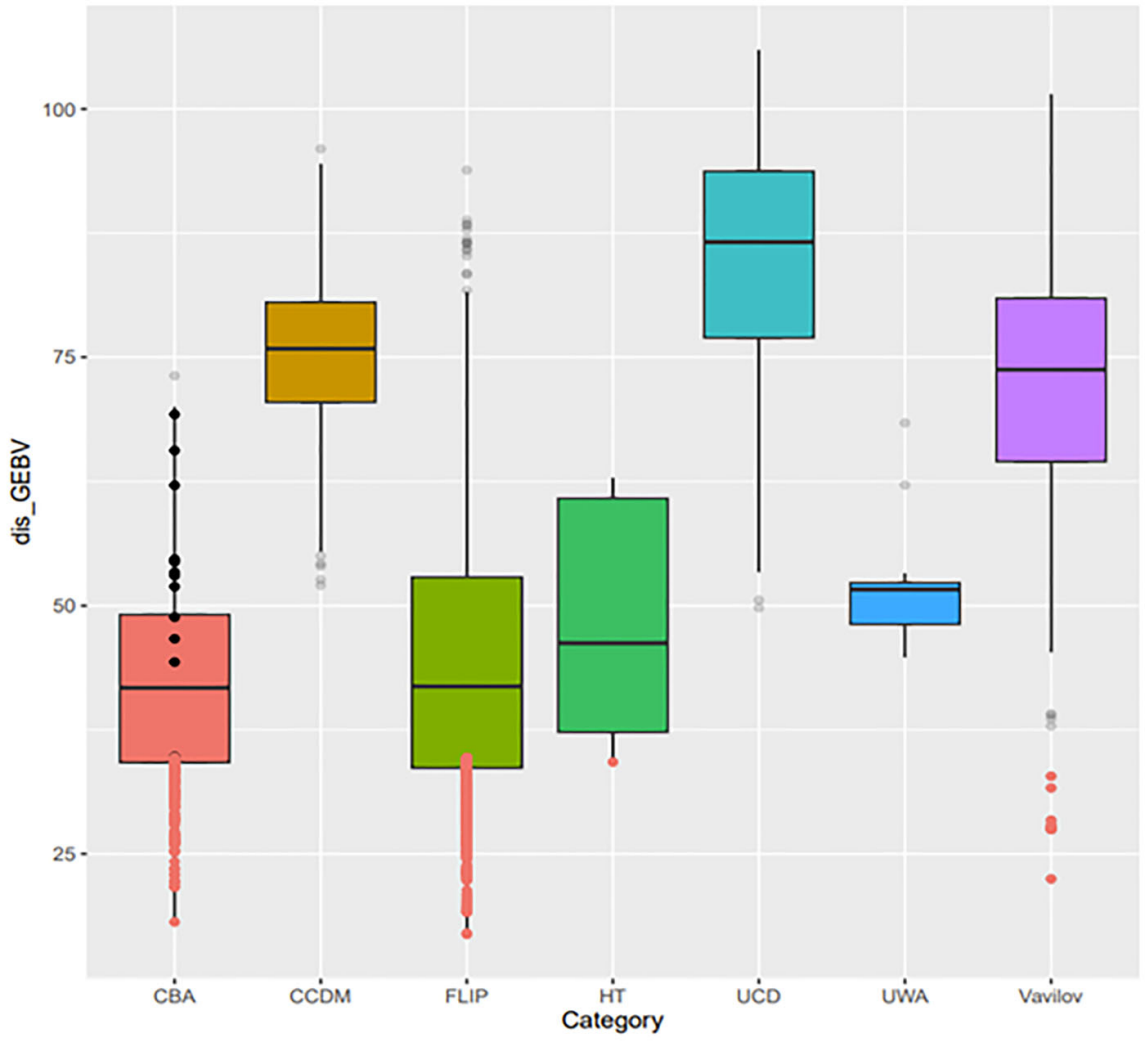
Distribution of genomic estimated breeding values (GEBVs) for stem breakage among different germplasm collections. The dots in red are the lines (*n* = 462) having more Ascochyta blight (AB) resistance than the released check varieties in terms of GEBV. The dots in bold black (Chickpea Breeding Australia [CBA] only) are the released check varieties.

GEBVs for stem breakage were estimated for all genotypes using a bivariate GBLUP model, where lower GEBVs indicate higher AB resistance. Note that 462 of the 2790 lines ranked better than the released check varieties. The distribution of GEBVs per germplasm collection is shown in Figure 3. Across the collections, individual CBA and FLIP lines (the red and the green boxplots in Figure 3) tended to show the lowest GEBVs, and hence increased AB resistance. While some lines from Vavilov also showed higher AB resistance compared to the released check varieties in terms of GEBVs. Details of GEBVs can be found in Supporting Information 1, except the materials from CBA due to confidential reasons.

## 4 DISCUSSION

The current study evaluated the potential for deploying GS to improve AB resistance in chickpea through exploiting qualitative and quantitative genome‐wide marker effects. The high genomic prediction accuracy achieved in this study validates the possibility of deploying GS as an alternative approach to improve AB resistance in chickpea. Earlier studies have identified dominant or recessive genes affecting AB resistance in chickpea (Farahani et al., 2019; Labdi et al., 2013; Singh & Reddy, 1989, 1991). To date, conventional breeding programs have focused on improving AB resistance through phenotypic selection. As a result, most released chickpea varieties offer only partial resistance to AB and can exhibit various responses to different isolates/strains of the pathogen (Chen & Muehlbauer, 2003; Ramen et al., 2022). Due to narrow genetic diversity in cultivated chickpea, new sources of AB resistance are often introgressed into breeding germplasm from diverse sources including landraces and wild relatives, which is time‐consuming. The ability of GS to rapidly accumulate both major and minor effect genes into a single genotype may offer a complementary approach to introgression for increasing AB resistance in chickpea.

### 4.1 Correlation between field and pot‐based screening

The genotypic responses to different isolates screened in the pot‐based assay exhibited high genetic correlations (>0.8), except for isolate TR8102 (0.04–0.27). One possible explanation is the smaller degree of overlap of lines (<10) between pot‐based assay in which TR8102 was only tested in 2016 and 2017 (Table S3). Consequently, the correlation between TR8102 and other isolates may have been underestimated. A similar positive correlation in response to isolates (c. 0.8) was reported by Pande et al. (2011). Overall, the high genetic correlations observed in our study support our raw data processing approach, which treated the response to different isolates as a single trait. The results suggest a genotype demonstrating durability to one isolate may also exhibit durability to other isolates. This highlights the potential of utilizing GS to breed genetically durable chickpea varieties capable of withstanding most, if not all, isolates.

High genetic correlation was observed between the field and the outdoor pot‐based screens. Field screening techniques for AB response are well developed and have been refined over the years through various experiments (Fanning et al., 2022; Pande et al., 2011; Reddy et al., 1980; Singh, 1981). These techniques involve interplanting susceptible spreader lines and may include dispersing naturally infected debris between rows as source of natural inoculum. In this study, the outdoor pot‐based facility offered a similar environment to the field environment leading to high genetic correlation between the two screening methods for adapted germplasm. Due to the high similarity between the two systems in terms of revealing genotypic responses to AB, only field data were used for QTL identification. The high genetic correlation also suggests breeders can use either screening systems to precisely identify and select lines having desirable responses to specific isolates. The high genetic correlation also holds significant promise for scaling phenotyping efforts for well adapted germplasm, particularly in breeding programs aimed at improving resistance to AB. It also provides new opportunities to employ high‐throughput phenotyping approaches using unmanned air vehicles in the field to reduce the labor and cost associated with traditional phenotyping.

### 4.2 Identification of major QTL for AB resistance

Six major QTL explaining 33% of the genetic variance for AB resistance were identified across five chromosomes (Ca1, Ca2, Ca3, Ca5, and Ca7). Previous studies have also reported major QTL for AB resistance (Flandez‐Galvez et al., 2003; Granato & Fritsche‐Neto, 2018; Iruela et al., 2006; Li et al., 2018; Ramen et al., 2022; Tar'an et al., 2007; Tekeoglu et al., 2002), notably on chromosome 4. For example, Deokar, Sagi, and Tar'an (2019) reported two QTL on Ca4, qAB4.1, and qAB4.2, conferring AB resistance. Similarly, Sudheesh et al. (2021) reported a single QTL on Ca4 conferring AB resistance from an interspecific cross between *C. arietinum* and *Cicer echinospermum*. In this study, only intermediate marker effects were observed on Ca4, which were below our threshold for declaring the presence of a major QTL. Multiple factors can contribute to the variability in reported QTL regions. Variation in the genetic backgrounds of the chickpea germplasm evaluated in different studies can result in the identification of distinct QTL. In Kottapalli et al. (2009), the QTL for AB resistance detected varied across crosses, with each cross having its unique genetic background (e.g., unique parental lines) within the mapping population. In addition, differences in pathogen virulence between studies and genotype‐by‐environment‐by‐management interactions (G × E × M) can lead to the detection of different resistance loci. The detection of six QTL in this study likely results from a combination of the large population size screened and its diversity, noting that it comprised collections capturing significant proportion of global chickpea diversity, as well as *C. arietinum* × *C. a. reticulatum* introgression lines. To the best of our knowledge, most QTL identified are novel to any published QTL, offering new sources of resistance for developing AB resistant chickpea varieties.

### 4.3 Genetic responses and genomic predictions

The AB responses observed in this study showed a high degree of heritability, with a narrow‐sense heritability of 0.82 for the field data and 0.69 for the pot‐based data. These estimates are consistent with previous studies. For example, high (c. 0.8) heritability for AB resistance was reported for half‐diallel crosses between *C. arietinum* and wild species of *C. reticulatum* and *C. echinospermum* (Danehloueipour et al., 2007; Labdi et al., 2015). Similarly, high heritability (0.67–0.85) was observed in reciprocal populations (e.g., two populations from different heterotic groups) for the trait (Lichtenzveig et al., 2002). The high narrow‐sense heritability observed in this study indicates that most of the trait variance is under genetic control, making it highly amenable to GS. This finding is further supported by the intermediate‐to‐high levels of genomic prediction accuracy observed across the different validation scenarios (Table 3). The small‐to‐moderate level of population differentiation (*Fst* = 0.197) found among overall population, in theory, can also facilitate the implementation of GS. These results demonstrate GS has the potential to be a reliable approach to guide breeding decisions for improving AB resistance in chickpea. To the best of our knowledge, no previous studies have evaluated GS for improving AB resistance in chickpea. The closest known study by Carpenter et al. (2018) explored genomic prediction accuracy for AB resistance in pea (*Pisum sativum* L.). In comparison to their findings, the accuracies achieved in our study are higher, primarily due to our use of a larger training population and a single AB pathogen affecting chickpea compared to field pea, where the disease in pea can be caused by a complex of three different pathogens.

Genomic prediction accuracy was high when using the field data to predict responses from the pot‐based data for well adapted germplasm and vice versa. This was expected given the high (0.91) genetic correlation between the field and the pot‐based screens. Also interesting was the intermediate to high level of accuracy observed when a genomic prediction model trained on one isolate was used to predict the response to another. This is likely due to that most AB isolates in Australia share common ancestry and low diversity of the test strains due to the presence of a single mating type (MAT1‐2; Bar et al., 2021). Therefore, isolates in Australia have similar genetic response, facilitating the process of using one isolate to predict another.

In addition, in terms of GEBVs, the study found that AB resistance was relatively absence in wild introgression lines (CCDM and UC Davis, Figure 3), while a comparatively wider range of resistance traits in cultivated introgression lines (e.g., CBA). This is consistent with the absence of natural selection for AB resistance in wild *Cicer* populations. AB is a disease driven by moisture and temperature (Moore et al., 2015). The high plant density and closed canopies that typified chickpea agriculture created local humidity within the canopy, consequently facilitating disease development. However, these conditions are absent from the populations in the wild that are normally in low plant density and small local population sizes. Thus, it is likely that AB occurred in agriculture would never reach epidemic proportions in the wild, and consequently, natural selection of AB is absent in the wild (McDonald & Stukenbrock, 2016). The high level of resistance observed in the commercial breeding lines from CBA was due to the enrichment of AB resistance introgressed from wild chickpea (Kristy Hobson, personal communication, 2024), with completely different sources of wild *Cicer* sources in their backgrounds compared to the ones from CCDM and UC Davis screened in this study.

### 4.4 From qualitative to quantitative—Implications for breeding

Currently, all Australian commercial chickpea varieties possess partial resistance to AB, being rated moderately susceptible or susceptible (GRDC, 2024). The susceptibility of these varieties to rapid changes in AB pathogen virulence makes the development of durable resistance a pressing need. Historically, large effect QTL identified from genetic mapping studies have been introgressed into breeding germplasm to develop AB‐resistant chickpea varieties. While numerous QTL have been identified to date (Flandez‐Galvez et al., 2003; Granato & Fritsche‐Neto, 2018; Iruela et al., 2006; Li et al., 2018; Ramen et al., 2022; Tar'an et al., 2007; Tekeoglu et al., 2002), the ongoing discovery of new QTL is constrained by the narrow diversity present within the gene pool of cultivated chickpea (Abbo et al., 2003; Admas et al., 2021; von Wettberg et al., 2018). This has led to the search for useful sources of resistance to AB in the wider chickpea gene pool including landraces and cross‐compatible annual *Cicer* relatives (Jha et al., 2022; Newman et al., 2021, etc.).

The observation in our study and by others that AB resistance in chickpea is a moderately complex trait, controlled by both minor and larger effect genes (Deokar, Sagi, & Tar'an, 2019; Sudheesh et al., 2021), places GS as a powerful alternative for breeding chickpea varieties with more durable AB resistance. By using genome‐wide markers to estimate genomic breeding values, GS allows for the identification of superior lines that possess both major and minor resistance genes. In our study, the cumulative effect of minor resistance genes explained 67% of the genetic variance for AB resistance. This suggests that considerable improvements in AB resistance can be achieved by accumulating minor effect genes, along with larger effect genes, into a single genotype. Applying GS will allow breeders to better exploit existing genetic variation within the cultivated gene pool, while simultaneously introgressing larger effect genes for AB resistance into new varieties. As found in the study, 462 out of 2790 lines had better AB resistance than released check varieties in terms of GEBVs (mainly from the collections of CBA, FLIP, and Vavilov, details see Atieno et al., 2024), revealing the potential of further improvement for AB resistance for the industry by targeted, GS‐predicted combination of germplasm from the panel.

## 5 CONCLUSION

The present study has demonstrated the potential of deploying GS to breed for AB resistance in chickpea. The intermediate‐to‐strong genomic prediction accuracy achieved in the study provides evidence in support of implementing GS to guide breeding decision of AB resistance. Compared to the conventional breeding strategy, a genomic approach utilizing GEBVs predicted by genome‐wide distributed markers would be more efficient for breeding for durable AB resistance, taking qualitative and quantitative marker effects into account. In addition, the six major QTL identified in the study are distant to any published QTL. Further study is required to assess the potential of the newly identified QTL as new sources of AB resistance for commercial chickpea in Australia.

## AUTHOR CONTRIBUTIONS

**Zibei Lin**: Conceptualization; data curation; formal analysis; investigation; methodology; project administration; resources; software; supervision; validation; visualization; writing—original draft; writing—review and editing. **Yongjun Li**: Conceptualization; data curation; investigation; methodology; resources; software; validation; visualization; writing—review and editing. **Adnan Riaz**: Data curation; formal analysis; software; writing—review and editing. **Shimna Sudheesh**: Resources; writing—review and editing. **Shahin Yazdifar**: Investigation; resources; writing—review and editing. **Judith Atieno**: Methodology; resources; writing—review and editing. **Sara Blake**: Investigation; resources; writing—review and editing. **Janine Croser**: Funding acquisition; resources; supervision; writing—review and editing. **Joshua Fanning**: Funding acquisition; resources; supervision; writing—review and editing. **Matthew J. Hayden**: Funding acquisition; supervision; writing—review and editing. **Sukhjiwan Kaur**: Conceptualization; funding acquisition; investigation; methodology; project administration; supervision; writing—original draft; writing—review and editing.

## CONFLICT OF INTEREST STATEMENT

The authors declare no conflicts of interest.

## Supporting information



Supplementary Table 1. Phenotyping details from the field trials and the pot‐based assay
Supplementary Table 2. Fixation indices (*Fst*) between populations
Supplementary Table 3. Numbers of overlapping lines between trials (lower diagonal) and numbers of lines per trials (diagonal) investigated from the raw phenotypes

Supplementary File 1. GEBVs evaluated for all genotypes, excluded CBA materials due to confidential reasons.

## Data Availability

All data generated or analyzed during this study are included in this published article (and its supplementary information files). https://doi.org/10.5061/dryad.j6q573nrk.

## References

[tpg270023-bib-0001] Abbo, S. , Berger, J. , & Turner, N. (2003). Viewpoint: Evolution of cultivated chickpea: Four bottlenecks limit diversity and constrain adaptation. Functional Plant Biology, 30(10), 1081–1087. 10.1071/FP03084 32689090

[tpg270023-bib-0002] Admas, S. , Tesfaye, K. , Haileselassie, T. , Shiferaw, E. , & Flynn, K. (2021). Genetic variability and population structure of Ethiopian chickpea (*Cicer arietinum* L.) germplasm. PLoS ONE, 16(11), e0260651. 10.1371/journal.pone.0260651 34843606 PMC8629288

[tpg270023-bib-0003] Atieno, J. , Davidson, J. , Blake, S. , Hayes, J. , Fanning, J. , Krysinska‐Kaczmarek, M. , Hobson, K. , Kaur, S. , Sutton, T. , & Croser, J. (2024). *Multi‐environment screening identifies novel sources of Ascochyta blight resistance in chickpea*. Manuscript in preparation.

[tpg270023-bib-0004] Bar, I. , Sambasivam, P. , Davidson, J. , Farfan‐Caceres, L. , Lee, R. , Hobson, K. , Moore, K. , & Ford, R. (2021). Current population structure and pathogenicity patterns of *Ascochyta rabiei* in Australia. Microbial Genomics, 7(7). 10.1099/mgen.0.000627 PMC8477395 34283013

[tpg270023-bib-0005] Breen, E. J. , MacLeod, I. M. , Ho, P. N. , Haile‐Mariam, M. , Pryce, J. E. , Thomas, C. D. , Daetwyler, H. D. , & Goddard, M. E. (2022). BayesR3 enables fast MCMC blocked processing for largescale multi‐trait genomic prediction and QTN mapping analysis. Communications Biology, 5, 661. 10.1038/s42003-022-03624-1 35790806 PMC9256732

[tpg270023-bib-0006] Browning, B. L. , & Browning, S. R. (2013). Improving the accuracy and efficiency of identity‐by‐descent detection in population data. Genetics, 194(2), 459–471. 10.1534/genetics.113.150029 23535385 PMC3664855

[tpg270023-bib-0007] Butler, D. G. , Cullis, B. R. , Gilmour, A. R. , Gogel, B. J. , & Thompson, R. (2018). ASReml‐R reference manual v4 . https://asreml.kb.vsni.co.uk/wp-content/uploads/sites/3/ASReml-R-Reference-Manual-4.2.pdf

[tpg270023-bib-0008] Carpenter, M. A. , Goulden, D. S. , Woods, C. J. , Thomson, S. J. , Kenel, F. , Frew, T. J. , Cooper, R. D. , & Timmerman‐Vaughan, G. M. (2018). Genomic selection for ascochyta blight resistance in pea. Frontiers in Plant Science, 9, 1878. 10.3389/fpls.2018.01878 30619430 PMC6306417

[tpg270023-bib-0009] Chen, W. , & Muehlbauer, F. (2003). An improved technique for virulence assay of ascochyta rabiei on chickpea. International Chickpea and Pigeonpea Newsletter, 10, 31–33.

[tpg270023-bib-0010] Crossa, J. , Pérez‐Rodríguez, P. , Cuevas, J. , Montesinos‐López, O. , Jarquín, D. , De Los Campos, G. , Burgueño, J. , González‐Camacho, J. M. , Pérez‐Elizalde, S. , Beyene, Y. , Dreisigacker, S. , Singh, R. , Zhang, X. , Gowda, M. , Roorkiwal, M. , Rutkoski, J. , & Varshney, R. K. (2017). Genomic selection in plant breeding: Methods, models, and perspectives. Trends in Plant Science, 22(11), 961–975. 10.1016/j.tplants.2017.08.011 28965742

[tpg270023-bib-0011] Daba, K. , Deokar, A. , Banniza, S. , Warkentin, T. D. , & Tar'an, B. (2016). QTL mapping of early flowering and resistance to ascochyta blight in chickpea. Genome, 59(6), 413–425. 10.1139/gen-2016-0036 27244453

[tpg270023-bib-0012] Danehloueipour, N. , Yan, G. , Clarke, H. J. , & Siddique, K. H. M. (2007). Diallel analyses reveal the genetic control of resistance to ascochyta blight in diverse chickpea and wild *Cicer* species. Euphytica, 154(1), 195–205. 10.1007/s10681-006-9287-0

[tpg270023-bib-0013] Deokar, A. , Sagi, M. , Daba, K. , & Tar'an, B. (2019). QTL sequencing strategy to map genomic regions associated with resistance to ascochyta blight in chickpea. Plant Biotechnology Journal, 17, 275–288. 10.1111/pbi.12964 29890030 PMC6330535

[tpg270023-bib-0014] Deokar, A. , Sagi, M. , & Tar'an, B. (2019). Genome‑wide SNP discovery for development of high‑density genetic map and QTL mapping of ascochyta blight resistance in chickpea (*Cicer arietinum* L.). Theoretical and Applied Genetics, 132, 1861–1872. 10.1007/s00122-019-03322-3 30879097 PMC6531409

[tpg270023-bib-0015] Fanning, J. , Brand, J. , Munoz Santa, I. , Mcdonald, L. , Taylor, J. , & Hollaway, G. (2022). Management of chickpea Ascochyta blight using fungicides and cultivar resistance improves grain yield, quality, and grower profitability. Frontiers in Plant Science, 13, 942220. 10.3389/fpls.2022.942220 36352886 PMC9638893

[tpg270023-bib-0016] Farahani, S. , Talebi, R. , Maleki, M. , Mehrabi, R. , & Kanouni, H. (2019). Pathogenic diversity of *Ascochyta rabiei* isolates and identification of resistance sources in core collection of chickpea germplasm. The Plant Pathology Journal, 35(4), 321–329. 10.5423/PPJ.OA.12.2018.0299 31481855 PMC6706013

[tpg270023-bib-0017] Flandez‐Galvez, H. , Ades, P. K. , Ford, R. , Pang, E. C. K. , & Taylor, P. W. J. (2003). QTL analysis for ascochyta blight resistance in an intraspecific population of chickpea (*Cicer arietinum* L.). Theoretical and Applied Genetics, 107, 1257–1265. 10.1007/s00122-003-1371-4 12928777

[tpg270023-bib-0018] Gebremedhin, A. , Li, Y. , Shunmugam, A. S. K. , Sudheesh, S. , Valipour‐Kahrood, H. , Hayden, M. J. , Rosewarne, G. M. , & Kaur, S. (2024). Genomic selection for target traits in the Australian lentil breeding program. Frontiers in Plant Science, 14, 1284781. 10.3389/fpls.2023.1284781 38235201 PMC10791954

[tpg270023-bib-0019] Ghaffari, P. , Talebi, R. , & Keshavarzi, F. (2014). Genetic diversity and geographical differentiation of Iranian landrace, cultivars, and exotic chickpea lines as revealed by morphological and microsatellite markers. Physiology and Molecular Biology of Plants, 20, 225–233. 10.1007/s12298-014-0223-9 24757326 PMC3988336

[tpg270023-bib-0020] Global Market Insight . (2023). Chickpeas market size—By product type, form, sales channel, application analysis, share, growth forecast, 2025–2034 . https://www.gminsights.com/industry‐analysis/chickpeas‐market#:~:text=Chickpeas%20Market%20size%20was%20valued,CAGR%20from%202023%20to%202032

[tpg270023-bib-0021] Granato, I. , & Fritsche‐Neto, R. (2018). R‐package ‘snpReady’ . https://cran.r‐project.org/web/packages/snpReady/snpReady.pdf

[tpg270023-bib-0022] GRDC . (2024). National variety trials . https://nvt.grdc.com.au/nvt‐disease‐ratings

[tpg270023-bib-0023] Iruela, M. , Rubio, J. , Barro, F. , Cubero, J. I. , Millán, T. , & Gil, J. (2006). Detection of two quantitative trait loci for resistance to ascochyta blight in an intra‐specific cross of chickpea (*Cicer arietinum* L.): Development of SCAR markers associated with resistance. Theoretical and Applied Genetics, 112, 278–287. 10.1007/s00122-005-0126-9 16328235

[tpg270023-bib-0024] Jamalabadi, J. G. , Saidi, A. , Karami, E. , Kharkesh, M. , & Talebi, R. (2013). Molecular mapping and characterization of genes governing time to flowering, seed weight, and plant height in an intraspecific genetic linkage map of chickpea *(Cicer arietinum*). Biochemical Genetics, 51, 387–397. 10.1007/s10528-013-9571-3 23371372

[tpg270023-bib-0025] Jha, U. , Sharma, K. , Nayyar, H. , Parida, S. , & Siddique, K. (2022). Breeding and genomics interventions for developing ascochyta blight resistant grain legumes. International Journal of Molecular Sciences, 23(4), 2217. 10.3390/ijms23042217 35216334 PMC8880496

[tpg270023-bib-0026] Jighly, A. , Hayden, M. , & Daetwyler, H. (2021). Integrating genomic selection with a genotype plus genotype x environment (GGE) model improves prediction accuracy and computational efficiency. Plant, Cell and Environment, 44, 3459–3470. 10.1111/pce.14145 34231236

[tpg270023-bib-0027] Kottapalli, P. , Gaur, P. M. , Katiyar, S. K. , Crouch, J. H. , Buhariwalla, H. K. , Pande, S. , & Gali, K. K. (2009). Mapping and validation of QTLs for resistance to an Indian isolate of Ascochyta blight pathogen in chickpea. Euphytica, 165, 79–88. 10.1007/s10681-008-9762-x

[tpg270023-bib-0028] Labdi, M. , Ghomari, S. , & Hamdi, S. (2015). Combining ability and gene action estimates of eight parent diallel crosses of chickpea for Ascochyta blight. Advances in Agriculture, 2015, 832597. 10.1155/2015/832597

[tpg270023-bib-0029] Labdi, M. , Malhotra, R. , Benzohra, I. E. , & Imtiaz, M. (2013). Inheritance of resistance to *Ascochyta rabiei* in 15 chickpea germplasm accessions. Plant Breeding, 132(2), 197–199. 10.1111/pbr.12038

[tpg270023-bib-0030] Li, Y. , Ruperao, P. , Batley, J. , Edwards, D. , Davidson, J. , Hobson, K. , & Sutton, T. (2018). Genome analysis identified novel candidate genes for Ascochyta blight resistance in chickpea using whole genome re‐sequencing data. Frontiers in Plant Science, 8, 359. 10.3389/fpls.2017.00359 PMC5355423 28367154

[tpg270023-bib-0031] Lichtenzveig, J. , Shtienberg, D. , Zhang, H. B. , Bonfil, D. J. , & Abbo, S. (2002). Biometric analyses of the inheritance of resistance to *Didymella rabiei* in chickpea. Phytopathology, 92, 417–423. 10.1094/PHYTO.2002.92.4.417 18942955

[tpg270023-bib-0032] Lin, Z. , Cogan, N. , Pembleton, L. , Spangenberg, G. , Forster, J. , Hayes, B. , & Daetwyler, H. (2016). Genetic gain and inbreeding from genomic selection in a simulated commercial breeding program for perennial ryegrass. The Plant Genome, 9(1), plantgenome2015.06.0046. 10.3835/plantgenome2015.06.0046 27898764

[tpg270023-bib-0033] Lin, Z. , Robinson, H. , Godoy, J. , Rattey, A. , Moody, D. , Mullan, D. , Keeble‐Gagnere, G. , Forrest, K. , Tibbits, J. , Hayden, M. J. , Daetwyler, H. , & Pino Del Carpio, D. (2021). Genomic prediction for grain yield using genotype‐by‐environment interaction clusters: A case study in a commercial barley breeding program. Crop Science, 61, 2323–2335. 10.1002/csc2.20460

[tpg270023-bib-0034] Markell, S. , Wise, K. , McKay, K. , Goswami, R. , & Gudmestad, N. (2008). Ascochyta blight of chickpea . https://www.ndsu.edu/pubweb/~goswami/Publications/Aschochyta%20Blight%20fact-sheet.pdf

[tpg270023-bib-0035] McDonald, B. , & Stukenbrock, E. (2016). Rapid emergence of pathogens inagro‐ecosystems: Global threats toagricultural sustainability andfood security. Philosophical Transactions of the Royal Society B: Biological Sciences, 371, 20160026. 10.1098/rstb.2016.0026 PMC5095548 28080995

[tpg270023-bib-0036] Moore, K. , Ryley, M. , Cumming, G. , & Jenkins, L. (2015). Chickpea: Ascochyta blight management (Australia Pulse Bulletin). Pulse Australia. http://www.pulseaus.com.au/growing‐pulses/bmp/chickpea/ascochyta‐blight

[tpg270023-bib-0037] Newman, T. , Jacques, S. , Grime, C. , Kamphuis, F. , Lee, R. , Berger, J. , & Kamphuis, L. (2021). Identification of novel sources of resistance to Ascochyta blight in a collection of wild *Cicer* accessions. Phytopathology, 111(2), 369–379. 10.1094/PHYTO-04-20-0137-R 32787627

[tpg270023-bib-0038] Pande, S. , Sharma, M. , Gaur, P. M. , Tripathi, S. , Kaur, L. , Basandrai, A. , Khan, T. , Gowda, C. L. L. , & Siddique, K. H. M (2011). Development of screening techniques and identification of new sources of resistance to Ascochyta blight disease of chickpea. Australasian Plant Pathology, 40(2), 149–156. 10.1007/s13313-010-0024-8

[tpg270023-bib-0039] Raman, R. , Warren, A. , Krysinska‐Kaczmarek, M. , Rohan, M. , Sharma, N. , Dron, N. , Davidson, J. , Moore, K. , & Hobson, K. (2022). Genome‐wide association analyses track genomic regions for resistance to *Ascochyta rabiei* in Australian chickpea breeding germplasm. Frontiers in Plant Science, 13, 877266. 10.3389/fpls.2022.877266 35665159 PMC9159299

[tpg270023-bib-0040] R‐Core‐Team . (2022). R: A language and environment for statistical computing. R Foundation for Statistical Computing. https://www.R‐project.org/

[tpg270023-bib-0041] Reddy, M. V. , Nene, Y. L. , & Singh, K. B. (1980). Field screening of chickpea for resistance to Ascochyta blight. International Chickpea Newsletter, 2, 13–15.

[tpg270023-bib-0042] Singh, K. B. (1981). Resistance in Chickpeas to *Ascochyta rabie* . Plant Disease, 65, 586–587. 10.1094/PD-65-586

[tpg270023-bib-0043] Singh, K. B. , & Reddy, M. V. (1989). Genetics of resistance to Ascochyta blight in four chickpea lines. Crop Science, 29(3), 657–659. 10.2135/cropsci1989.0011183X002900030022x

[tpg270023-bib-0044] Singh, K. B. , & Reddy, M. V. (1991). Advances in disease‐resistance breeding in chickpea. Advances in Agronomy, 45, 191–222.

[tpg270023-bib-0045] Stephens, A. , Lombardi, M. , Cogan, N. O. I. , Forster, J. W. , Hobson, K. , Materne, M. , & Kaur, S. (2013). Genetic marker discovery, intraspecific linkage map construction and quantitative trait locus analysis of ascochyta blight resistance in chickpea (*Cicer arietinum* L.). Molecular Breed, 33, 297–313. 10.1007/s11032-013-9950-9

[tpg270023-bib-0046] Sudheesh, S. , Kahrood, H. V. , Braich, S. , Dron, N. , Hobson, K. , Cogan, N. O. I. , & Kaur, S. (2021). Application of genomics approaches for the improvement in Ascochyta blight resistance in chickpea. Agronomy, 11, 1937. 10.3390/agronomy11101937

[tpg270023-bib-0047] Tar'an, B. , Warkentin, T. D. , Tullu, A. , & Vandenberg, A. (2007). Genetic mapping of ascochyta blight resistance in chickpea (*Cicer arietinum* L.) using a simple sequence repeat linkage map. Genome, 50(1), 26–34. 10.1139/g06-137 17546068

[tpg270023-bib-0048] Tekeoglu, M. , Rajesh, P. , & Muehlbauer, F. (2002). Integration of sequence tagged microsatellite sites to the chickpea genetic map. Theoretical and Applied Genetics, 105, 847–854. 10.1007/s00122-002-0993-2 12582909

[tpg270023-bib-0049] VanRaden, P. M. (2008). Efficient methods to compute genomic predictions. Journal of Dairy Science, 91(11), 4414–4423. 10.3168/jds.2007-0980 18946147

[tpg270023-bib-0050] Varshney, R. K. , Song, C. , Saxena, R. K. , Azam, S. , Yu, S. , Sharpe, A. G. , Cannon, S. , Baek, J. , Rosen, B. D. , Tar'an, B. , Millan, T. , Zhang, X. , Ramsay, L. D. , Iwata, A. , Wang, Y. , Nelson, W. , Farmer, A. D. , Gaur, P. M. , Soderlund, C. , & Cook, D. R. (2013). Draft genome sequence of chickpea (*Cicer arietinum*) provides a resource for trait improvement. Nature Biotechnology, 31, 240–248. 10.1038/nbt.2491 23354103

[tpg270023-bib-0051] von Wettberg, E. J. B. , Chang, P. L. , Başdemir, F. , Carrasquila‐Garcia, N. , Korbu, L. B. , Moenga, S. M. , Bedada, G. , Greenlon, A. , Moriuchi, K. S. , Singh, V. , Cordeiro, M. A. , Noujdina, N. V. , Dinegde, K. N. , Shah Sani, S. G. A. , Getahun, T. , Vance, L. , Bergmann, E. , Lindsay, D. , Mamo, B. E. , & Cook, D. R. (2018). Ecology and genomics of an important crop wild relative as a prelude to agricultural innovation. Nature Communications, 9(1), 649. 10.1038/s41467-018-02867-z PMC5811434 29440741

[tpg270023-bib-0052] Wickham, H. (2016). ggplot2: Elegant graphics for data analysis. Springer‐Verlag New York. https://ggplot2.tidyverse.org

[tpg270023-bib-0053] Windhausen, V. S. , Atlin, G. N. , Hickey, J. M. , Crossa, J. , Jannink, J.‐L. , Sorrells, M. E. , Raman, B. , Cairns, J. E. , Tarekegne, A. , Semagn, K. , Beyene, Y. , Grudloyma, P. , Technow, F. , Riedelsheimer, C. , & Melchinger, A. E. (2012). Effectiveness of genomic prediction of maize hybrid performance in different breeding populations and environments. G3: Genes| Genomes| Genetics, 2(11), 1427–1436. 10.1534/g3.112.003699 23173094 PMC3484673

