# Supplementary material for: Assessing the utility of genomic selection to breed for durable Ascochyta blight resistance in chickpea
Source: Plant Genome. 2025 Mar 31;18(2):e70023. doi: 10.1002/tpg2.70023 (PMC11958870; doi:10.1002/tpg2.70023)
Supplement: Supplementary file 1 [file TPG2-18-e70023-s001.docx]

**Supplementary Table 1. Phenotyping details from the field trials and the pot-based assay**

| **Trial type** | **Trial** | **Isolate** | **No. of observations** |
| --- | --- | --- | --- |
| Field | 2020-Field | Mixed | 722 |
|  | 2021-Field | Mixed | 987 |
|  | 2022-Field | Mixed | 724 |
| Pot-based | 2016-Ref | 16CUR018, TR8102 | 207 |
|  | 2016-UWA | FT13092-1 | 21 |
|  | 2017-FLIP | 16CUR018, TR8102 | 67 |
|  | 2018-FLIP | F17191-1, TR9571 | 37 |
|  | 2019-FLIP | 16CUR018 | 182 |
|  | 2019-CBA | 16CUR018, TR9571 | 212 |
|  | 2020-FLIP | 16CUR018 | 42 |
|  | 2021-FLIP | 16CUR018, AR0128 | 228 |
|  | 2022-Coreset | AR0226 | 284 |
|  | 2022-NorthernCoreset | 16CUR018 | 607 |

**Supplementary Table 2. Fixation indices (*Fst*) between populations**

|  | FLIP | CBA | Vavilov | UWA | UC Davis | CCDM |
| --- | --- | --- | --- | --- | --- | --- |
| CBA | 0.297 | - | - | - | - | - |
| Vavilov | 0.242 | 0.107 | - | - | - | - |
| UWA | 0.328 | 0.199 | 0.043 | - | - | - |
| UC Davis | 0.282 | 0.128 | 0.024 | 0.000 | - | - |
| CCDM | 0.272 | 0.149 | 0.066 | 0.106 | 0.064 | - |
| HT | 0.330 | 0.201 | 0.043 | 0.306 | 0.000 | 0.107 |

**Supplementary Table 3. Numbers of overlapping lines between trials (lower diagonal) and numbers of lines per trials (diagonal) investigated from the raw phenotypes**

|  | **2016 Ref** | **2016 UWA** | **2017 FLIPs** | **2018 FLIPs** | **2019 FLIPs** | **2019 CBA** | **2020 FLIPs** | **2021 FLIPs** | **2022 Coreset** | **2022 Northern Coreset** | **2020 Field** | **2021 Field** | **2022 Field** |
| --- | --- | --- | --- | --- | --- | --- | --- | --- | --- | --- | --- | --- | --- |
| **2016 Ref** | 207 |  |  |  |  |  |  |  |  |  |  |  |  |
| **2016 UWA** | 4 | 21 |  |  |  |  |  |  |  |  |  |  |  |
| **2017 FLIPs** | 2 | 1 | 67 |  |  |  |  |  |  |  |  |  |  |
| **2018 FLIPs** | 1 | 1 | 1 | 37 |  |  |  |  |  |  |  |  |  |
| **2019 FLIPs** | 1 | 1 | 66 | 37 | 182 |  |  |  |  |  |  |  |  |
| **2019 CBA** | 5 | 6 | 2 | 1 | 1 | 212 |  |  |  |  |  |  |  |
| **2020 FLIPs** | 1 | 1 | 1 | 1 | 16 | 1 | 42 |  |  |  |  |  |  |
| **2021 FLIPs** | 1 | 14 | 44 | 20 | 98 | 2 | 12 | 228 |  |  |  |  |  |
| **2022 Coreset** | 1 | 8 | 44 | 15 | 98 | 59 | 16 | 176 | 607 |  |  |  |  |
| **2022 Northern Coreset** | 2 | 10 | 44 | 16 | 98 | 61 | 17 | 177 | 282 | 284 |  |  |  |
| **2020 Field** | 5 | 8 | 4 | 1 | 21 | 211 | 19 | 10 | 84 | 84 | 722 |  |  |
| **2021 Field** | 5 | 18 | 66 | 35 | 180 | 12 | 38 | 178 | 175 | 174 | 75 | 987 |  |
| **2022 Field** | 4 | 14 | 42 | 13 | 90 | 74 | 18 | 169 | 531 | 243 | 94 | 167 | 724 |

**Supplementary File 1, GEBVs evaluated for all genotypes, excluded CBA materials due to confidential reasons.**
