# Supplementary material for: Assessing the utility of genomic selection to breed for durable Ascochyta blight resistance in chickpea
Source: Plant Genome. 2025 Mar 31;18(2):e70023. doi: 10.1002/tpg2.70023 (PMC11958870; doi:10.1002/tpg2.70023)
Supplement: Supplementary file 1 — Supplementary Table 1. Phenotyping details from the field trials and the pot‐based assay Supplementary Table 2. Fixation indices (Fst) between populations Supplementary Table 3. Numbers of overlapping lines between trials (lower diagonal) and numbers of lines per trials (diagonal) investigated from the raw phenotypes [file TPG2-18-e70023-s001.docx]

**Supplementary Table 1. Phenotyping details from the field trials and the pot-based assay**

| **Trial type** | **Trial** | **Isolate** | **No. of observations** |
| --- | --- | --- | --- |
| Field | 2020-Field | Mixed | 722 |
|  | 2021-Field | Mixed | 987 |
|  | 2022-Field | Mixed | 724 |
| Pot-based | 2016-Ref | 16CUR018, TR8102 | 207 |
|  | 2016-UWA | FT13092-1 | 21 |
|  | 2017-FLIP | 16CUR018, TR8102 | 67 |
|  | 2018-FLIP | F17191-1, TR9571 | 37 |
|  | 2019-FLIP | 16CUR018 | 182 |
|  | 2019-CBA | 16CUR018, TR9571 | 212 |
|  | 2020-FLIP | 16CUR018 | 42 |
|  | 2021-FLIP | 16CUR018, AR0128 | 228 |
|  | 2022-Coreset | AR0226 | 284 |
|  | 2022-NorthernCoreset | 16CUR018 | 607 |

**Supplementary Table 2. Fixation indices (*Fst*) between populations**

|  | FLIP | CBA | Vavilov | UWA | UC Davis | CCDM |
| --- | --- | --- | --- | --- | --- | --- |
| CBA | 0.297 | - | - | - | - | - |
| Vavilov | 0.242 | 0.107 | - | - | - | - |
| UWA | 0.328 | 0.199 | 0.043 | - | - | - |
| UC Davis | 0.282 | 0.128 | 0.024 | 0.000 | - | - |
| CCDM | 0.272 | 0.149 | 0.066 | 0.106 | 0.064 | - |
| HT | 0.330 | 0.201 | 0.043 | 0.306 | 0.000 | 0.107 |

**Supplementary Table 3. Numbers of overlapping lines between trials (lower diagonal) and numbers of lines per trials (diagonal) investigated from the raw phenotypes**

|  | **2016 Ref** | **2016 UWA** | **2017 FLIPs** | **2018 FLIPs** | **2019 FLIPs** | **2019 CBA** | **2020 FLIPs** | **2021 FLIPs** | **2022 Coreset** | **2022 Northern Coreset** | **2020 Field** | **2021 Field** | **2022 Field** |
| --- | --- | --- | --- | --- | --- | --- | --- | --- | --- | --- | --- | --- | --- |
| **2016 Ref** | 207 |  |  |  |  |  |  |  |  |  |  |  |  |
| **2016 UWA** | 4 | 21 |  |  |  |  |  |  |  |  |  |  |  |
| **2017 FLIPs** | 2 | 1 | 67 |  |  |  |  |  |  |  |  |  |  |
| **2018 FLIPs** | 1 | 1 | 1 | 37 |  |  |  |  |  |  |  |  |  |
| **2019 FLIPs** | 1 | 1 | 66 | 37 | 182 |  |  |  |  |  |  |  |  |
| **2019 CBA** | 5 | 6 | 2 | 1 | 1 | 212 |  |  |  |  |  |  |  |
| **2020 FLIPs** | 1 | 1 | 1 | 1 | 16 | 1 | 42 |  |  |  |  |  |  |
| **2021 FLIPs** | 1 | 14 | 44 | 20 | 98 | 2 | 12 | 228 |  |  |  |  |  |
| **2022 Coreset** | 1 | 8 | 44 | 15 | 98 | 59 | 16 | 176 | 607 |  |  |  |  |
| **2022 Northern Coreset** | 2 | 10 | 44 | 16 | 98 | 61 | 17 | 177 | 282 | 284 |  |  |  |
| **2020 Field** | 5 | 8 | 4 | 1 | 21 | 211 | 19 | 10 | 84 | 84 | 722 |  |  |
| **2021 Field** | 5 | 18 | 66 | 35 | 180 | 12 | 38 | 178 | 175 | 174 | 75 | 987 |  |
| **2022 Field** | 4 | 14 | 42 | 13 | 90 | 74 | 18 | 169 | 531 | 243 | 94 | 167 | 724 |

**Supplementary File 1, GEBVs evaluated for all genotypes, excluded CBA materials due to confidential reasons.**
